# First difference mode interferometer demonstration for a high-bandwidth Electro-Optic Beam Position Monitor

**DOI:** 10.1038/s41598-025-08786-6

**Published:** 2025-12-06

**Authors:** A. Arteche, S. M. Gibson, A. Schloegelhofer, T. E. Levens, T. Lefèvre

**Affiliations:** 1 Physics Department, John Adams Institute for Accelerator Science at Royal Holloway, University of London, Egham Hill, Egham, TW20 0EX UK; 2 CERN, CH-1211 Geneva 23, Switzerland

**Keywords:** Experimental particle physics, Photonic devices, Design, synthesis and processing

## Abstract

This work presents, for the first time, the experimental demonstration of the differential-field detection mode $\Delta$ as a key component in the ongoing development of a novel interferometric Electro-Optic Beam Position Monitor (EO-BPM), capable of high-bandwidth monitoring of $1\,\textrm{ns}$-long HL-LHC ultra-relativistic proton bunches. Through the utilization of an innovative fibre-coupled Mach-Zehnder detection scheme, in its first experimental implementation, this study proves that the new field-focusing pickup design engineered to facilitate long-distance and high-bandwidth single-pass detection can deliver a sub-millimetric detection resolution while keeping an ultrafast time response below the HL-LHC goal of $50\,\textrm{ps}$. The transverse-position and time-resolution capability of the system were addressed at HiRadMat and CLEAR beamlines, respectively. The transverse position study was performed within a $\pm 20\,\textrm{mm}$ range at $3\,\textrm{GHz}$ acquisition bandwidth for SPS-like parameters ($4\sigma \approx 1.5\,\textrm{ns}\, \& \,1.2\times 10^{11}p^{+}$), whereas a $33\,\textrm{GHz}$ response was achieved by detecting short CLEAR electron bunches ($4\sigma \approx 20\,\textrm{ps}$). In addition, the stability of the signals acquired under high levels of back-scattering radiation also proves that, due to the optical nature of the device, the EO-BPM differential-field mode is unaffected, and therefore very suitable for such environments.

## Introduction

### The Electro-Optic Beam Position Monitor

The High Luminosity Large Hadron Collider (HL-LHC) program constitutes a major upgrade to increase the luminosity by an order of magnitude. The project includes the installation of transverse deflecting RF devices called crab cavities at the interaction regions. The objective is to optimize the geometric overlap between counter-propagating beams by inducing a tilt that makes the bunches cross head-on at the collision point^1^. The procedure also compensates for the deflection, thus returning the bunches to their original orientation after the collision. Head-Tail (HT) monitors are a type of diagnostic that was extensively used during the crab cavity prototyping phase at the CERN-SPS to detect any head-tail difference after compensation^2^. However, electromagnetic striplines offer a limited bandwidth ($2-3\,\textrm{GHz}$), below expected specifications ($6-10\,\textrm{GHz}$)^3^.

Several technologies have been suggested to address the challenge of limited bandwidth. Cherenkov Diffraction Radiation Monitors have shown an extremely high-bandwidth response, however, their effectiveness in this application is constrained by their too high low-frequency cut-off ($>1\,\textrm{GHz}$)^4,5^. Alternatively, EO diagnostics have primarily been based on quadratic effect techniques for bunch length monitoring and beam profiling of short electron beams^6^. To ensure the detection, those EO schemes often require frequency decoding techniques and placing the EO crystal a few millimetres away from the beam. There also exists a high-bandwidth EO technology that, thanks to the fast time responses of commercial modulators, is employed as arrival monitors in electron LINACS. Nevertheless, this hybrid approach has proved not suitable to provide a faithful time-profile replica of the beam that is crucial in intra-bunch diagnostics^7^. Conversely, there is more scarcity in the literature regarding linear-effect EO crystals, but notably, an alternative linear sampling EO-BPM concept for electron beams was proposed by K. Hunt-Stone et al.^8^.

In this context, the interferometric Electro-Optic Beam Position Monitor (EO-BPM) has been proposed for various high-bandwidth applications^9–11^. The EO-BPM concept relies on the linear ultra-fast response of EO crystals of Lithium Niobate (LN) that are embedded in the core of the pickup. The propagating field of a passing relativistic bunch is encoded onto a laser optical beam as it travels perpendicular to the field across the crystal. The intra-bunch transverse reconstruction follows the standard $\Delta /\Sigma$ approach, using three independent signals: one differential signal ($\Delta$) from the field difference across opposing pickup crystals, and two lateral signals whose sum provides the normalization term ($\Sigma$). In this work, we focus on the experimental demonstration of the differential field $\Delta$-mode, which is optically encoded through a single interferometric channel– enabling higher position resolution than conventional BPMs that rely on electronic subtraction.

Based on this technique, the EO-BPM goal is to deliver beam-induced optical signals with a time resolution below $50\,\textrm{ps}$, which implies operational bandwidths of at least 6–$10\,\textrm{GHz}$^9^, while the turn-by-turn transverse resolution goal for the HL-LHC is set at $10\,\mu \textrm{m}$ for the position of the proton bunch peak.

### Novel waveguide field-focusing pickup

**Fig. 1:**
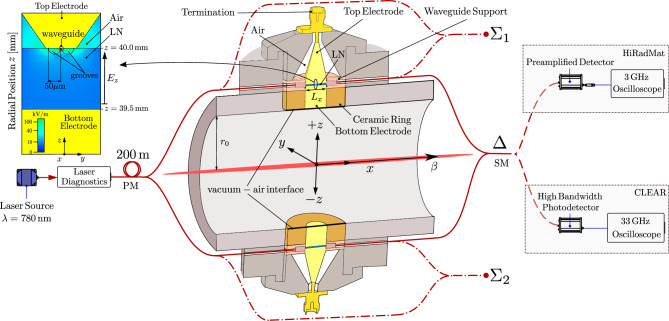
Schematic diagram of the full EO-BPM system, which is based on three Mach-Zehnder interferometers in combination with two waveguide field-focusing pickups: the $\Delta$-mode interferometer measuring the differential electric field across opposing crystal pickups, and two side interferometers with unmodulated arms that measure individual field components to retrieve the normalization term ($\Sigma = \Sigma _1 + \Sigma _2$). The bottom electrode is centrally mounted on a dielectric ceramic alumina ring that defines the vacuum-to-air interface, ensuring that the LN waveguide remains in atmospheric conditions, thus preserving its optical performance.

Here we report, for the first time, the successful performance of an EO diagnostic for long-distance and single-shot acquisition of long proton beams ($4\sigma>1\,\textrm{ns}$). The design effectively addresses several obstacles compared to some of the existing EO techniques mentioned earlier for electron machines, where: (i) the Coulomb field is typically three orders of magnitude lower than those for short electron bunches due to the significantly longer bunch length; (ii) the crystals are not a few millimetres away from the beam, as it happens for EO bunch length monitors of short electron bunches^6^, but assembled inside pickups that are located as far as the LHC pipe radius $(r_0=30.5\,\textrm{mm})$. Therefore, coupling a sufficient Coulomb field responsible for the signal into the pickup is far more challenging in this system than for most EO diagnostics for electron beams.

In late 2016, a prototype EO pickup delivered the first averaged signals of a circulating proton bunch obtained by EO means, implying the detection of low beam-induced modulating fields below $1\,\mathrm {kV/m}$^12–14^. Several upgrades towards a more compact and robust interferometer model were systematically integrated, improving the signal strength by incorporating a floating electrode to enhance the modulating field by a factor $2-3$^13,15–17^.

Following that proof-of-concept, we present the results obtained with the latest vacuum-compatible pickup version that is shown in Fig. 1: The LN crystal is held between a floating bottom electrode facing the beam and a top conical-shaped electrode that, together with the surrounding metallic housing, forms an approximate $50\,\Omega$ transmission line. This line is connected via an SMA coaxial connector to a broadband $50\,\Omega$ terminator with an $18\,\textrm{GHz}$ bandwidth. Despite the design, mechanical tolerances may introduce impedance mismatches at high frequencies. The cylindrical bottom electrode is mounted at the centre of a dielectric ceramic mica ring support, and an additional ceramic piece is used to precisely hold the LN waveguide in place between the electrodes. Although a characterization in in-air facilities is presented, the pickup is designed so that a vacuum-to-air interface between the beam pipe and the optical components is established at the level of the mica-supported bottom electrode assembly, ensuring that the waveguide crystal remains in air and is thus protected from vacuum-induced optical degradation. This configuration has already been successfully implemented in an in-vacuum installation at the CERN SPS in 2023^18^.

However, the main novelty is that now the optically active part is confined within a $L_x=9\,\textrm{mm}$ long ridge waveguide formed at the top of the crystal. As depicted on the top left corner of Fig. 1, the waveguide is located in between two $50\,\mathrm {\mu m}$-radius in-air grooves that are carefully engineered, so in combination with the refined tipped top electrode design, the image field vector lines are precisely directed across the waveguide volume.

**Fig. 2:**
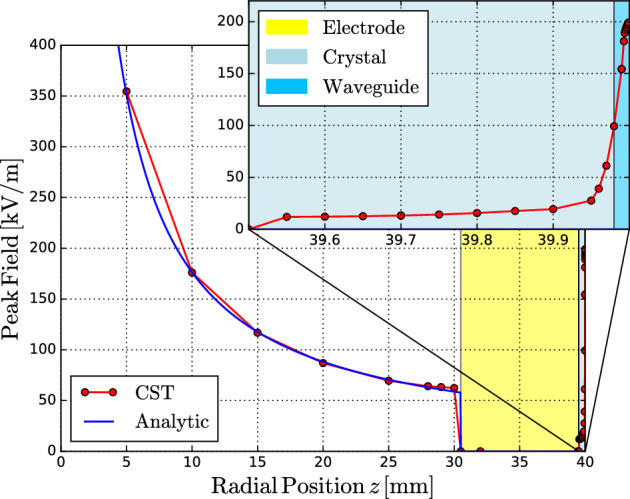
CST simulation outcome of the peak electric field along the radial distance *z* and through the EO-BPM pickup, including the LN crystal and waveguide. The results illustrate the field-focusing mechanism under HL-LHC nominal beam parameters.

Fig. 2 shows the results of numerical simulations performed using the Particle Studio suite package of Computer Simulation Technology (CST) software^19^. Given a Gaussian LHC nominal bunch ($4\sigma =1\,\textrm{ns} \, \& \, \,1.15\times 10^{11}\,p^+$), the propagating peak field decays with $z^{-1}$ inside the pipe until reaching the floating electrode at the edge. The electric field drops to zero at the copper floating bottom electrode, as no electric field exists inside a conductor due to boundary conditions. The role of this electrode is not to accumulate charge, but rather to transmit an image charge across the EO crystal sample. Then the original hot-spot mechanism delivers a largely magnified time-profile replica of the propagating Coulomb field inside the waveguide, reaching a peak field value above $190\,\mathrm {kV/m}$, i.e., at least a 200 increase factor with respect to the prototype model described earlier.

### Mach-Zehnder interferometric detection

Fig. 1 shows how a continuous wave optical beam emerging from a $\lambda =780\,\textrm{nm}$ tunable laser source, is power-amplified and carried over $200\,\textrm{m}$ via Polarisation-Maintaining (PM) fibre, from the surface to the system arrangement in the underground beamline. The beam is then split in the tunnel to create a Mach-Zehnder-$\Delta$ interferometer mounted on a $61\,\textrm{mm}$ diameter pipe. Each of the optical paths is linearly (vertically) polarised ([0, 0, 1]) and coupled to the LN *z*-cut ridge waveguide formed on top of a $0.5\,\textrm{mm}$ thick substrate along the $x$-direction. The optomechanical design has been engineered so that the modulating field $E_z$ co-propagates to improve phase matching with the optical beam to guarantee the required bandwidth. The birefringence-induced property, known as the Pockels effect, modifies the refractive index $n_z$ linearly with the electric field $E_z$. As result, a field-dependent phase retardation $\phi (E_z)$ is induced in the outgoing optical beam^20^:

1 $$\begin{aligned} \phi (E_z)=\phi _0+\phi (E_z)=\phi _0+\frac{\pi }{\lambda }\,r_{33}\,n_z^3\,L_x\,E_z \, , \end{aligned}$$

where $L_x$ is the waveguide length in the optical beam direction, $\phi _{0}=2\,\pi \,n_z\, L_x/\lambda$ is the phase offset in absence of field $(E_z=0)$ due to the natural birefringence of LN, and $r_{33}$ is the linear EO coefficient.

In combination with a highly efficient pickup, the $\Delta$-mode detection scheme replicates a Mach-Zehnder interferometer, just as shown in the central layout of Fig. 1: two optical paths through coplanar waveguide pickups are combined interferometrically at the interaction point $\Delta$, where two optical outputs are delivered: one in-phase and one anti-phase. The extent of the phase difference $\Delta \phi _{\Delta }$ at a given output in $\Delta$ can be derived from Eq. 1 as a function of the modulating field difference $\Delta E_z(t)$ between opposite pickups as

2 $$\begin{aligned} \Delta \phi _{\Delta }\left[ \Delta E_z(t)\right] =\frac{\pi }{\lambda }\,r_{33}\,n_z^3\,\,L_x\,\Delta E_z(t) \, , \end{aligned}$$

assuming $\phi _0=0$. The detection sensitivity is determined by a characteristic parameter named $E_{\pi }$, which is defined as the extent of $\Delta E_z$ required to transit from an in-phase constructive state to a non-transmission output, i.e., $\Delta \phi =\pi$, then

3 $$\begin{aligned} E_{\pi }=\Delta E_z= \frac{\lambda}{r_{33}\,n_z^3\, L_x } , \end{aligned}$$

where $r_{33}=30.8\,\mathrm {pm/V}$ and $n_z\simeq2.18$^21^; $E_{\pi }$ must be maximised but given that $L_x$ is limited to $9\,\textrm{mm}$ to guarantee the bandwidth performance and $\lambda$ is set at $780\,\textrm{nm}$ to prevent the photorefractive effect^22^, then $E_{\pi }=272\,\mathrm {kV/m}$, which is in the same order as $E_z$ for the optimised pickup, according to the CST simulations. With this detection scheme, the EO-BPM $\Delta$-mode signal is only produced when the beam is off-centre, delivering a field-difference profile that is proportional to both position and intensity, and is optically encoded on a single channel. This approach can potentially offer superior position resolution compared to traditional BPMs or head-tail striplines, where the beam position is reconstructed by electronically subtracting signals from opposite pickups.

## EO-BPM characterisation campaign

### Beam position studies at HiRadMat

The first interferometric EO-BPM based on this fully fibre-coupled waveguide model was propitiously installed at the HiRadMat facility^23,24^. Since it is an SPS extraction line, the proton beam parameters are very similar to those injected in the LHC. The EO-BPM system was assembled within an in-air pipe section and mounted on a stepping-motor translation stage, allowing precise movement relative to the particle beam trajectory over a $\pm 20\,\textrm{mm}$ range. Positive offsets ($+z$) indicate the beam approaches one pickup, while negative values ($-z$) indicate it approaches the opposite^25^. In-air transverse resolution tests were performed parasitically for single shots below nominal ($\sim 0.7\times 10^{11}\,\mathrm {p^+}$) while some targets near the EO-BPM were heavily irradiated.

**Fig. 3:**
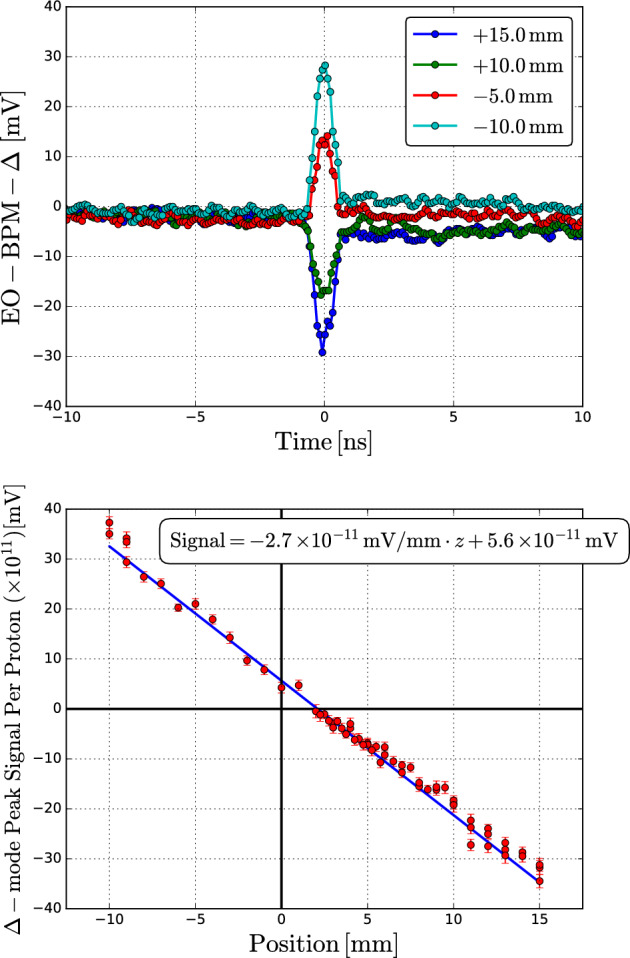
HiRadMat results: (top) collection of EO-BPM $\Delta$-mode traces acquired at $3\,\textrm{GHz}$ bandwidth for various transverse offsets; (bottom) peak signal normalised per proton against translation stage position.

The beam-induced optical signal produced at $\Delta$ is then conveyed about $20\,\textrm{m}$ by standard Single Mode (SM) fibre to the adjacent tunnel, where it was recorded by an acquisition system comprised by a $10\,\textrm{GHz}$ pre-amplified detector AC-coupled to an 8-bit, $10\,\textrm{GSPS}$, $3\,\textrm{GHz}$-bandwidth oscilloscope. As the laser source was tunable, it enabled setting the desired working point $\phi _0(\lambda )$ by adjusting the wavelength. After each trace acquisition, the wavelength was then slightly shifted–with the assistance of a feedback diagnostic system–until the crossing point between the two interferometer outputs, phase and antiphase, was found. This point corresponds to the $\pi /2$ phase offset, where both outputs are equally sensitive. This procedure ensured consistent working point conditions for all data points.

Fig. 3 shows a subset of single proton beam traces taken at HiRadMat during the campaign. The presence of a small pedestal following the main pulse is an artefact attributed to the use of a preamplified detector, likely caused by residual baseline recovery effects in the amplifier stage. The peak value obtained from each trace fit versus the translation stage position *z* is also presented for the entire collection of acquisitions. Each peak value has been normalised by the number of protons per bunch, as measured by a Beam Current Transformer (BCT) installed next to the EO-BPM. This represents, to our knowledge, the first set of bunch-by-bunch measurements ever obtained optically by applying an EO Mach-Zehnder scheme between opposite pickups. The results show good linearity over a wide range of positions, with a transverse resolution $\sigma _z$ estimated as the ratio between the residual standard deviation $\sigma _{\text {residual}}$ and the slope of the linear fit *m*, evaluated within the $\pm 3\,\textrm{mm}$ region around the offset, i.e., $\sigma _z = \sigma _{\text {residual}} / m$. Using $\sigma _{\text {residual}} = 1.1\,\textrm{mV}$ and a gradient of $2.7\,\mathrm {mV/mm}$ in that interval, a position resolution of $400\,\mathrm {\mu m}$ is obtained. This value does not take into account shot-by-shot beam position jitters nor laser optical power fluctuations. Consequently, the position resolution that shall be achieved in the LHC is expected to be largely improved.

Importantly, the $3\,\textrm{GHz}$ bandwidth acquisition is comparable to the state-of-the-art, i.e. the HT stripline monitors^3^. Noteworthy, a perfectly balanced system should lead to a null signal at $z=0$. A discernible offset is noticeable in the dataset, most likely due to an asymmetry that was identified during the pickup assembly process and could be further optimised.

### Radiation tolerance studies at HiRadMat

**Fig. 4:**
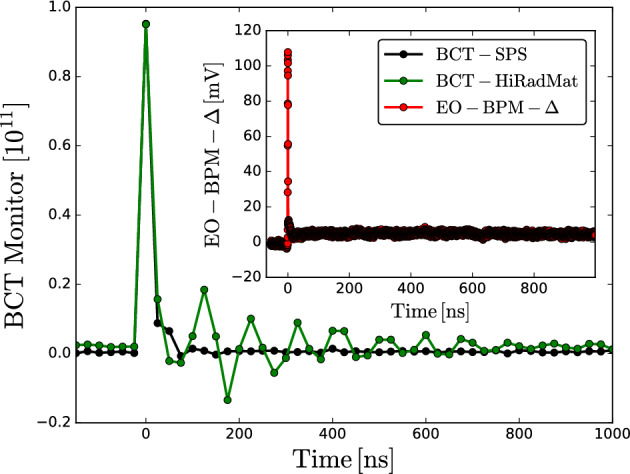
Acquisition of a single proton bunch passing by a BCT located in the SPS ring, compared with the same bunch extracted at HiRadMat for a second BCT and interferometric EO-BPM $\Delta$-mode, both of which were exposed to the back-scattering radiation of a graphite-copper target.

The campaign included characterisation studies to determine the suitability of the interferometric EO-BPM as a diagnostic option for harsh radiation environments. Fig. 4 shows the signals induced by the same nominal passing beam ($\sim 10^{11}\,\textrm{p}^+$) registered by three different diagnostics: Two BCTs, one located in the SPS ring and the other at HiRadMat–the latter previously mentioned for signal normalisation–along with the EO-BPM.

The SPS BCT is located in the SPS ring and thus delivers a standard unperturbed single-shot detection. During Hiradmat operation, the same beam, extracted from the SPS, impacts on a graphite-copper target located at the end of the line, and the BCT installed in the beamline is exposed to heavy back-scattering radiation, causing a significant distortion in the readout. The extent of the after-beam noise typically depends on the thickness and material type of the target. Nevertheless, even though the EO-BPM was also located next to the HiRadMat BCT, a few metres away from the target, it delivered a non-distorted signal, indicating an insensitivity to beam-induced radiation background and hadronic showers from the dump. However, while the EO-BPM showed no signal degradation during short-term exposure at HiRadMat, the potential long-term effects of radiation on the optical fiber have not been assessed in detail. In particular, radiation-induced darkening could impact optical transmission over extended operation. This aspect remains to be investigated in future dedicated studies. Nonetheless, since the optical components of the pickup are located outside the vacuum, they could potentially be replaced more easily in case of radiation-induced damage.

### Bandwidth characterisation studies at CLEAR

Following the transverse measurements obtained at HiRadMat, the same in-air EO-BPM test bench was moved to CLEAR^26^ for a data-taking campaign to demonstrate the time resolution goal of $50\,\textrm{ps}$ required for intra-bunch detection over a $1\,\textrm{ns}$ HL-LHC proton bunch. The CLEAR facility is a $200\,\textrm{MeV}$ electron LINAC at CERN that operates with bunch lengths ranging from $\sigma = 1\,\textrm{ps}$ to $5\,\textrm{ps}$ and single-bunch charge up to $1.5\,\textrm{nC}$. Such short electron bunches are ideal for evaluating the EO-BPM system’s response and ensuring it fulfils the temporal resolution criteria.

**Fig. 5:**
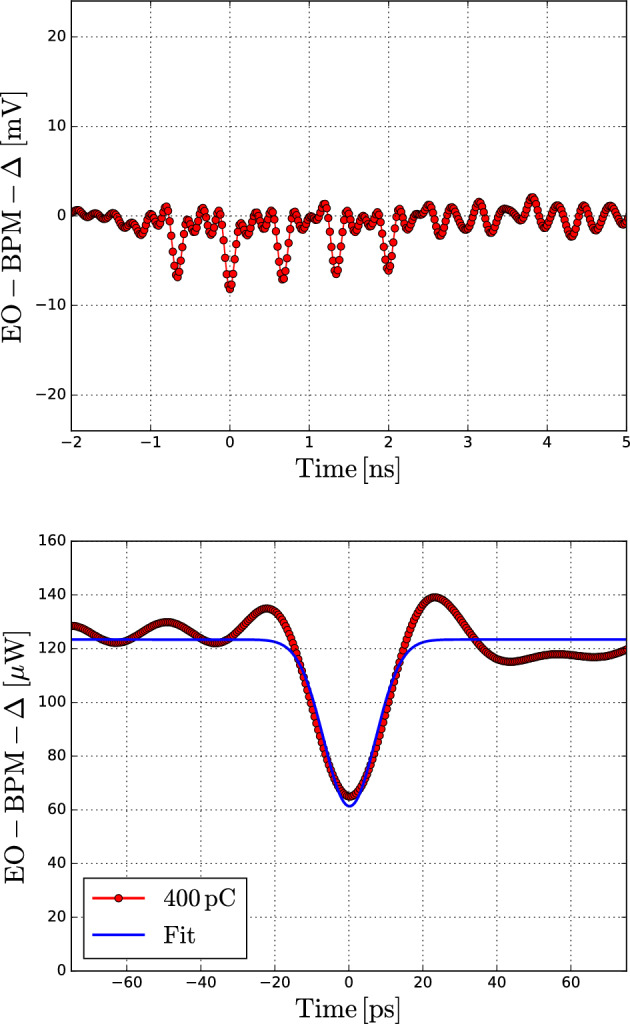
Bandwidth characterisation tests at CLEAR: (top) $5\,\textrm{GHz}$ acquisition of an electron bunch train spaced by $666\,\textrm{ps}$; (bottom) single-shot acquisition at $33\,\textrm{GHz}$ bandwidth of a $400\,\textrm{pC}$ and $\sigma \sim 5\,\textrm{ps}$ long CLEAR electron bunch.

Fig. 5 shows a bunch train modulation read through the same $10\,\textrm{GHz}$ pre-amplified detector but this time the signal was acquired by a $5\,\textrm{GHz}$ oscilloscope. The bunch repetition rate of $1.5\,\textrm{GHz}$ leads to a bunch spacing of $666\,\textrm{ps}$. The interferometric offset $\phi _0$ was set at maximum transmission so the signals showed a negative sign (eq. 1). Each signal corresponds to an actual $\sigma \sim 5\,\textrm{ps}$ long electron bunch but it appears widened due to the limited $5\,\textrm{GHz}$ bandwidth.

Further measurements were taken with an upgraded detection system. The optical modulation was connected to a dedicated $33\,\textrm{GHz}$ oscilloscope with an integrated photodetector that allowed for direct optical readout from the SM outcoming fibre (Fig. 1). This system feature could acquire the intensity-modulated signal together with the optical DC baseline, allowing for online monitoring of the working point.

Fig. 5 also shows an example of a $\sigma \sim 5\, \textrm{ps}$ single-shot electron bunch that was detected with the ultrafast $33\,\textrm{GHz}$ setup. Once again, the interferometric point was set at a maximum transmission of $\sim 120\,\mathrm {\mu W}$ DC power reaching the detector, thus the beam-induced modulation exhibits a negative sign. The Gaussian $\sigma$ obtained from the fitting of $6.9\,\textrm{ps}$ is compatible with the slight broadening expected due to the $33\,\textrm{GHz}$ upper limit capacity of the upgraded detection system.

This proves that the optomechanical design is capable of at least delivering a $33\,\textrm{GHz}$ bandwidth response. The time resolution of the interferometric EO-BPM is therefore well within the $<50\,\textrm{ps}$ specification required for the HL-LHC measurement of $4\sigma =1\,\textrm{ns}$ bunches.

## Conclusion

For the first time, an electro-optic Mach-Zehnder detection scheme between two opposite pickups has successfully been used to measure, with very high bandwidth, both $4\sigma>1\,\textrm{ns}$ proton bunches with submillimetric transverse resolution, and also $4\sigma \sim 20$ electron bunches. In comparison with other EO diagnostics, the present detection scheme is fully fibre-coupled, providing a compact, vacuum-compatible, robust, and easy-to-operate system. The bandwidth achieved in this design, up to $33\,\textrm{GHz}$, could be further optimised by simply improving the acquisition system, as the intrinsic pickup bandwidth has not yet been reached. Not only that, an even faster time response could be met by adapting the EO crystal length. Compared to any electromagnetic BPM technology, this detector offers a low cut-off frequency, allowing the measurements of the temporal profile of the beam with no distortion. Moreover, the system was successfully tested under high-radiation conditions and operated in close proximity to a high-power beam target. The measured resolution of $400\,\mathrm {\mu m}$ remains far from the ambitious $10\,\mathrm {\mu m}$ target defined for intra-bunch position diagnostics at the HL-LHC; however, this result was obtained under sub-nominal bunch charge conditions and with a non-optimised acquisition system, leaving considerable room for improvement. This innovative design represents an important milestone towards robust, high-bandwidth beam position monitoring techniques, that would find applications for intra-bunch motion or bunch length monitoring in hadron facilities, but also in electron facilities for bunch-by-bunch beam position monitoring.

## Data Availability

The datasets generated and analysed during the current study are stored in CERN’s internal repositories. Access can be granted on reasonable request.
